# Stocking up on Fish Mox: a Systematic Analysis of Cultural Narratives about Self-medicating in Online Forums

**DOI:** 10.3934/publichealth.2017.5.430

**Published:** 2017-09-29

**Authors:** Rebecca Howes-Mischel

**Affiliations:** Department of Sociology and Anthropology, James Madison University, Harrisonburg, VA, USA

**Keywords:** cultural narratives, self-medication, health inequality, populist expertise, authoritative knowledge, health narrative, digital cultures, disaster preparation, medical anthropology

## Abstract

This study is a systematic review of cultural narratives that drive American belief in the value and efficacy of stocking up on fish antibiotics for human consumption. Popularized by “doomsday prepper” forums and survivalist medical professionals' online videos, this narrative suggests that in some scenarios humans may benefit from such treatments—even as they note its contraindication to mainstream public health advice. Discussions in crowd-sourcing forums however, reveal that in practice Americans are using them as a form of home remedy to treat routine infections without missing work or to make up for gaps in insurance coverage. This article argues for greater attention to what makes it plausible and reasonable to treat human conditions with animal medications. It suggests that public health initiatives should address such decisions as emerging from a rational analysis of social and economic conditions rather than dismissing such practices as dangerous to population and individual health outcomes. As social scientists of medicine have long argued, collective narratives about health and medicine illustrate deeply the broader contexts in which communities understand and experience bodily state and shape how communities interact with public health institutions and respond to medical expertise. This study surveys online discussions about “fish mox” to show how participants contest medical expertise and promote a more distributed form of populist expertise. As such, consuming fish mox is both panacea for health inequality and a critique of health institutions for perpetrating such stratification.

## 1. Introduction

### 1.1. “How to use fish mox (to treat your sick fish of course)” [1]

The title of this blog post by “Dr. Bone”—the *nom de plume* of Joseph Alton, a retired doctor described as one of the leading experts of medical disaster preparedness—reflects a common rhetorical framing of online discussions about “fish mox”^1^ (a veterinary equivalent of amoxicillin). While its literal meaning is that fish mox is clearly for fish—signaling an awareness of public health norms—this is undercut as the article goes on to explain why it still may be a good strategy to stockpile supplements in case of future social or environmental collapse. Online discussions about medical preparedness credit U.S. Special Forces with popularizing fish mox use, and describe it as a rational form of self-medication when reliable medical facilities are inaccessible.^2^ Along with food, skills, and weapons, medical supplies are key to preparing for inevitable disaster. Yet medical providers are generally loathe to prescribe large amounts of antibiotics for unspecified future use (not to mention insurance companies' resistance to approving such costs). Thus, disaster prepping expert sites like Dr. Bone's Doom and Bloom or that of Patriot Nurse cautiously recommend stock piling fish mox instead—but only for consumption when one will (presumably) not be able to get to a doctor or obtain drugs. Trusted experts in these virtual communities thus promote stocking up on fish mox in anticipation of future disaster, even as they note its contraindication to mainstream public health advice and caution against using veterinary equivalents in so-called normal times.

Yet, systematic analysis of the tens of thousands of Google-indexed hits for queries such as “fish mox for humans”, “fish mox reviews”, and “fish mox safety” reveals that such cautions and future orientation fall away in more populist online deliberations. Amazon reviews, crowd-sourcing forums, and first-person blogs suggest that, in practice, Americans are self-medicating with these products to treat routine infections and either purposely bypassing medical institutions or making up for gaps in insurance coverage. While there is scant data about the prevalence of fish mox consumption (as opposed to talk about consumption), this practice should matter to public health practitioners given concerns about product safety as well as the risks of misdiagnosis, incorrect dosing, drug interactions, side-effects, and increased drug resistance globally [2].

Rather than advocating that healthcare practitioners simply dismiss these practices as dangerous to population and individual health outcomes, this article argues for greater attention to what makes it plausible and reasonable to treat human conditions with animal medications. Anthropological scholarship about survivalist cultures and online health-related decision-making [3] indicates that individuals draw on deeply familiar core beliefs to anticipate future catastrophes. This article extends that analysis to consider how online discussions of fish mox consumption transect both "survivalist or “disaster preparation” (commonly referred to as “prepper”) and non-prepper online discussions about health and precarity. Rather than approach these as distinct communities, I demonstrate that narratives and practices about future survival are instead mobilized to address and critique current health economies within online communities that include both an explicit survivalist ethos and those that do not. Close attention to the consistent patterning of such rhetorical framing and substantive content of cultural narratives about fish mox reveals foundational logics that implicate a broader spectrum of health-related beliefs emerging from deeply-rooted experiences of institutional distrust and socio-economic precarity.

The multiple variables (cultural and economic) that influence peoples' decisions to consume fish mox require successful health education interventions to do more than caution against this practice. As social scientists of medicine have long argued, collective narratives about health and medicine illustrate deeply held cultural logics about the broader contexts in which communities understand and experience bodily state—beliefs that shape how communities interact with public health institutions and medical expertise and that constitute multiple forms of what Brigitte Jordan terms “authoritative knowledge” [4]. Drawing on Briggs' framework of “communicability” [5] and Epstein's construct of “lay expertise” [6], I show that discourses about fish mox constitute a kind of counterhegemonic narrative whose challenge to the legitimacy of medical professionals as healing professionals is supported by experiences of political and economic crisis. Thus participants draw on prepper discussions of medical plausibility to express their alienation from health institutions and instead legitimate populist expertise. This research adds to recent scholarship that contextualizes public health refusals within extant social relations [7] and suggests that convincing people that fish mox consumption is disadvantageous requires health professionals to engage the authoritative narratives that support its use.

### 1.2. Populist expertise and authoritative knowledge

Medical anthropologists demonstrate the importance to public health institutions of taking cultural logics seriously for both pragmatic and epistemological reasons. First, taking them as well-structured examples of collective knowledge rather than superstition or misinformation improves the efficacy of public health institutions' cross-cultural or cross-community outreach. Second, foundational narratives and health-related logics constitute communities' fundamental and self-evident assumptions about the relationship between individual wellbeing and their broader socio-political, economic, and globalized contexts. Just as Good argues that the language of medicine (and by extension public health) is a “rich cultural language, linked to a highly specialized version of reality and system of social relations,” [8] narratives about the efficacy of fish mox express knowledge about a collective experience of reality and the relationship between health and stratified social relations.

Rather than privileging any knowledge as an *a priori* and stable reflection of the natural world, Jordan's construct of “authoritative knowledge” [4] points to the iterative power dynamics involved in accomplishing such certainty. This highlights the importance of institutional legitimation to render certain beliefs as empirical fact and attends to the terms through which collectives may argue for alternatively legitimate conclusions. As such, this article centers on what Charles Briggs terms “spheres of communicability” [5]: the networks and media through which information is produced, circulates, and is legitimated as authoritative knowledge. Such constructions of expertise both shape and reflect how we experience particular cultural worlds. Briggs' work shows that narrative accounts do more than define a situation, they also delineate *how* to understand phenomena credibly and the kinds of individuals authorized to present them as such. The normative model of communicability in medical and public health institutions relies on education, credentials, and social legitimacy. Narratives about consuming fish mox, however, constitute a counter narrative that shifts legitimacy away from experts to a populace by contesting how information about safety is deemed credible. Attention to communicability thus offers a framework for understanding how competing and dominant narratives about health beliefs circulate and interact to actively construct knowledge about the topic at hand, as well as about the relationship between different stakeholders and such information. That is, what makes knowledge authoritative.

Scholarship on the growth of rumors or “uncredible” knowledge illustrates that their communicability relies on aligning cultural beliefs with authoritative worldviews and previous experiences—in effect, a kind of confirmation bias. This is, more generally, the process through which observable data is transformed into authoritative knowledge by expert institutions. Thus, attention to narratives about knowledge that has not been granted this “fact” status—knowledge instead deemed “false”—illustrates alternative theories about the content and significance of observational data. To give an example, Scheper-Hughes argues for taking seriously the traction of rumors about body snatching and organ thefts that she found circulating within impoverished Brazilian communities (and which scholars have documented throughout the world) [9]. Rather than assessing the frequency of such occurrences by tracking them down and comparing official records to community accounts, she asks what makes them plausible and valid to communities globally—even, or especially, in the face of public campaigns that characterize them as disinformation. Relying on descriptive rather than prescriptive analysis, she shows that both expert and populist information rely on people's stories and narrative data presentations, but that only the former appear as official or legitimate accounts. She shows that the accounts dismissed by public agencies are grounded in, and rendered plausible through, lived experiences of how the bodies of poor people are more often exploited than cared for. Thus, while she does not conclude that shantytown residents' organs are literally at risk for systematic theft, she shows that at the level of metaphor this narrative is existentially true.

Similarly, Briggs and Mantini-Briggs [10] show that Venezuelan public health institutions' and Warao communities' explanations for a cholera outbreak in the Orinoco Delta 1992–1993 reveal how each relies on distorted beliefs about the other. Official narratives stressed the importance of hygienic practices and blamed “maladjusted” or “unmodern” cultural practices for the outbreak; indigenous communities instead blamed multinational corporations for conspiring with the government to dump poisonous materials in their region. Briggs and Mantini Briggs demonstrate that while neither narrative is empirically correct, the former constitutes a kind of authoritative truth claim, and the latter is easily dismissed as conspiracy theorizing. Yet, close examination shows that both accounts rely on “an abundance of readily observable details with secret knowledge of nefarious linkages. The result is a hypercoherent explanation that yields only one conclusion” [10]. As such, the Warao claims illustrate the logical extension of prior experiences of violence associated with Trinidadian trade incursions into the region and American oil extraction and constitute a rather sophisticated awareness of their marginal status in global health politics. As in Scheper-Hughes' work, taking non-expert narratives seriously shows that they are grounded in communities' experiences of their natural, social, and political worlds, and reveal a complex theory of power rooted in their worldviews. Drawing on these literatures, this analysis focuses on narratives institutionally classified as uncredible or risible to consider how they are instead illustrative of an alterative engagement with observable data.

Non-expert narratives about science, bodies, and health that are accepted as authoritative knowledge constitute a possible challenge to powerful institutions' authorizing power. As Epstein's classic examination of HIV treatment activism revealed, activists transformed themselves into participants in knowledge production in the absence of commitment from the medical establishment [6]. Activists generated crowd-sourced data through rigorous experimentation to establish their legitimacy as experts and ultimately influenced the course of institutional scientific knowledge production. Focusing on how such claims to expertise or official knowledge are *accomplished* thus shows how individuals collectively legitimize counterhegemonic beliefs by attaching them to institutions, practices, or discourses that are already deemed credible, influential, and trusted.

I draw on this schema to analyze how consumers of fish mox assert the legitimacy of crowd-sourced and evidence-based experimentation to challenge public health institutions by labeling the former more legitimate and trustworthy than the latter. This is in line with Reich's scholarship about motherhood and vaccine refusal [11], which traces how mothers use discourses of evidence-based rationality to contest official public health rationales. Narratives in favor of fish mox rely on the same foundational logics as public health professionals—this makes them powerful. Such narratives illustrate a form of “populist expertise” that values crowd-sourced and community-based experiential knowledge in direct opposition to socially authorized institutions.

## 2. Methods and Materials: Fish mox online

Americans increasingly use the Internet for health-related information [12],[13] and use it to form new support networks [14]. In particular, people with difficulty accessing health services due to underinsurance or travel time are more likely to turn to the Internet for assistance with health information [15],[16]. Online forums thus offer a window into the way communities assess and contest health-related information as authoritative knowledge. This qualitative discourse analysis is based on observations in publicly available digital prepper communities and associated online forums that reference fish mox consumption.^3^ While anthropological research about online communities still face questions about the boundaries of such communities, they constitute venues in which individuals come together to share information and in doing so build a common sensibility [17]. As such, these online communities produce and reproduce cultural beliefs as participants share information and develop local forms of expertise premised on shared experience and foundational worldviews.

Data were collected using iterative keyword searches to identify key expert figures in the medical preparedness community—and then through further social network analysis, through searching for discussions about antibiotics and fish mox on popular survivalist discussion boards and in online consumer reviews. The online spaces roughly fall into four categories: (1) pet supply sales sites that only advertise products; (2) pet supply sales sites with narratives about fish mox for human consumption (this includes Amazon.com); (3) prepper or survivalist sites that feature medical or otherwise “expert” guidance; (4) and online crowd-sourcing forums that include both neutral and explicit survivalist focus. This is in addition to more general health sites that warn the public away from consuming fish mox or report on the phenomenon. Here, I draw on analysis of 20 blogs and 3 public-discussion boards (including the heavily trafficked and popular survivalistboards.com). All material was originally posted publicly, however, in line with internet research ethical considerations and in light of the sensitivity of this content I have removed users' identifying information. Narratives about fish mox were coded using inductive open-coding methods and systematically analyzed for content themes reflective of underlying cultural beliefs. The majority of narratives were published between 2009 and 2016, although a 2002 letter to the New England Journal of Medicine from Pentagon doctors indicates that fish mox has been a popular medical strategy in the U.S. Special Forces for much longer [18]. Narratives about efficacy and safety (and desirability) fall into two broad categories: future-oriented cautious stockpiling and present consumption in place of human-grade and medically dispensed pharmaceuticals. As I discuss below, these are not two distinct orientations toward fish mox and there is an overlap between these communities. Instead, as individuals draw on cautionary advice about drastic scenarios to rationalize contemporary consumption, they illustrate shared cultural beliefs.

## 3. Fish Mox Narratives

There are three interrelated narratives about fish mox that individuals draw on to construct plausible rationales for self-medicating consumption: manufacturers' product descriptions, prepper experts' analysis of how fish mox fits into an uncertain future, and populist descriptions of the challenges of contemporary health economics. I briefly describe the significant characteristics of each below.

### 3.1. Selling fish mox

Fish mox is a broad-spectrum antibiotic that generally comes in 250 mg or 500 mg dose tablets or capsules (as well as bulk powder form) and is intended to treat common bacterial infections in ponds and aquariums. Unlike analogous treatments for dogs and cats, it does not require a prescription and can be purchased from pet supply stores (although some major companies will no longer stock them due to concerns about human consumption). Manufacturers' product descriptions and answers to Frequently Asked Questions (FAQ) caution that this treatment is “not for human use” and that treatment should be limited to ornamental fish rather fish destined for human consumption. Yet, discussions of safety and purity standards emphasize that these antibiotics are exactly the same as those dispensed from pharmacies for human use. These product descriptions also stress that antibiotics for fish and for humans are produced in the same plants and held to the same purity and quality standards established by the United States Pharmacopia (USP) overseen by the FDA. Rather than what's inside the bottle, they suggest that the difference in treatments is in the label “for fish use only,” which is required by law and unrelated to safety or dosage standard. These narratives are simultaneously legal disclaimers to avoid liability for off-label use, and powerful arguments that the difference between fish mox and amoxicillin for humans is one of relationship to legal and regulatory establishments rather than intrinsic difference in the substance of the treatment. For the purposes of this analysis, whether or not fish mox and human-grade pharmaceuticals are equivalent in composition, quality, or safety is immaterial. Instead, I am interested in the way narratives about this relationship to the official regulatory apparatus illustrate underlying logics about expertise. Unsurprisingly, this hedging is central to the argument survivalist consumers make about the benefits of stocking up on fish mox, with one site pointing to the legal disclaimers (described above) as evidence that the distinction between veterinary-grade and human-grade supplements is one of liability rather than the safety or quality of material substance.

### 3.2. Prepping for the future

Fish mox solves one of the conundrums facing preppers: how to ensure an adequate supply of broad-spectrum antibiotics after SHTF (“shit hits the fan”). Preppers work in the present to prepare to survive a future disaster or social collapse, which they are certain of will happen but uncertain what form it will take. Characterized by a belief in the instability of our current sociopolitical moment, they engage in probabilistic speculation and future-oriented consumption [3]. They reflect diverse consumption practices (from high-end bunkers to self-sufficiency workshops to stockpiles of basic necessities) that reflect a broad range of possible disaster scenarios. While news accounts of prepping communities emphasize that it is a “multi-billion-dollar” industry, in practice prepping is a set of consumption practices motivated by core values of future-oriented skepticism that cut across income levels. In general these values include mistrust of centralized governmental institutions and trust instead in individuals' responsibility to provide for themselves as their families (both as a moral stance and in anticipation of social breakdown). Health-related discussions reflect these core beliefs as well as the practicalities of living in a state of perpetual contingency [3]. They also point to an overlap between survivalists focused on the future and those who see the current moment as also requiring such self-sufficient survival skills due to institutional failures in the public safety net. Fish mox's use as a solution to the absence of functioning public health institutions is a practical demonstration of such beliefs and bolsters support for pharmaceutical self-sufficiency. Beyond the confines of expert blogs, discussions in prepper forums expose the overlap between communities focused on future precarity and those experiencing it currently. Crowd-sourcing narratives in discussion forums both rely on these writers' medical credentials and extrapolate from future worst-case scenarios to contemporary ones—casting skepticism at public health institutions in the process. Prepper blogs thus seem to be an origin point for the popularization of fish mox consumption beyond future-oriented prepper discussion by providing justification both for its probable safety and its use as critique.

### 3.3. Surviving late capitalism

In survivalist forums, individuals present an argument that future hedging and cautions to only use fish mox “when modern medical technology and resources are unavailable” misses the fact that currently such technology and resources are already inaccessible for many communities—that the future moment that mainstream speculates about is the contemporary one for rural and impoverished communities. In this vein, fish mox as “survival medicine” has clear and present uses.

“My fish came down with a nasty case of bronchitis and sinusitis just before Christmas, but her health insurance doesn't kick in until the first of the year. So she couldn't go to a fish doctor because she only makes minimum wage at the aquarium, and a trip to the fish emergency room would have put her in debt so far she wouldn't be able to get out. So she tapped on the edge of her tank with her sick little fin and blew bubbles in Morse code to ask me to order these for her. They worked great! She is now bronchitis and sinusitis-free, and she only had to miss one day of work at the aquarium. She thanked me in bubble Morse code, and said she would use them only when absolutely necessary, in order to avoid creating superfishbugs (Amazon review December 2016)”.

As this tongue-in-cheek review on Amazon.com for Fish Mox Forte makes clear, the decision to consume animal grade antibiotics is connected to larger stressors produced by the political economy of American healthcare. Without health insurance and working a low-wage job the Amazon reviewer frames this decision as their only option and as a carefully considered one. They both recognize that this practice officially framed as a discredited or ill-advised one and present themselves as a careful and rational consumer. Reviews for fish mox on Amazon are generally characterized by this “winking” rhetoric, which pays lip service to the questionable legality of selling/consuming animal-grade antibiotics for human consumption while still arguing for its benefit to attenuate the stressors of low-wage and underinsured household economics.^4^ They will only use the pills “when absolutely necessary,” implicitly, in much the same way as medical professionals.

Individuals in impoverished communities or without access to health institutions experience an increasingly unbalanced and humiliating world and draw on a range of cultural resources to make sense of the irrationality of such gross injustices. It is unsurprising that, for some, prepping for an uncertain future offers a sense of control over ones' survival that is missing from their contemporary experience. This is not to characterize all impoverished communities as reliant on survivalist resources, but rather to note how prepping communities' narratives of self-reliance offer resources for those who feel left behind by official institutions. Closely related, some survivalists articulate a deep skepticism that institutions they experience as having failed them—medical in this case—actually have an interest in protecting them. This skepticism leads people to scoff at warnings from government regulatory and health agencies cautioning against fish mox on the basis of safety. A post in a discussion on drugs.com is emblematic of that perspective:

“On the labels of the aquatic antibiotics it says ‘Not intended for human use’ because it is REQUIRED TO. It's a law, they only have to put that to cover themselves and that's how it sells so cheap, because it's [sic] ‘intended’ for fish. I get tonsillitis upwards of 3–5 times a year (can't yet afford a surgery) and I don't have the money to go to a doctor every time I need antibiotics for it. So this is a life saver (drugs.com October 2016, emphasis in original).”

Here, the consumer characterizes safety regulations as in the service of legal rather than public health caution and therefore uncredible.

Finally, some people seek to explain the humiliation and injustice of having to rely on fish mox through narratives that reflect an expectation that health is an individual responsibility (rather than a public good). They illustrate the strong focus on personal responsibility that saturates the survivalist community and echo classically neoliberal constructions of health as a matter of individual and disciplined choices [19],[20]. As one writer concludes about her decision to finally resort to using fish mox to treat increasingly debilitating tooth pain^5^.

“Since it happened to me, I feel there is something unethical about me being forced into taking fish medicine because of my poor financial luck. I did something wrong in life, apparently, to end up here. Business school, or nursing school, anything would have offered more security than my liberal arts education. I just dove in to this path without thinking at all about health care and insurance coverage. This was young and silly of me. I forgot that I live in America, where we have to look out for ourselves, and only ourselves. That's why I'm taking the fish meds. Because for every warning I read against taking the drugs, I wondered if the doctor or vet was being paid to say it might not be safe. Their arguments didn't stand up, except with big, scary adjectives used as props (Blue Lake Review, March 2015).”

Shame here is reframed to bemoan this individual's vocational choices in a social and political context in which such choices are literally the matter of life and death. The self-identified underemployed college instructor frames her decision to use fish mox as the result of the escalating financial precarity related in having neither stable income nor insurance coverage. Ultimately her narrative is an indictment of her own “bad choices” only insofar as American healthcare and insurance rely on individuals' economic maximization. It echoes what Rose describes as the alignment between “somatic ethics” and “the spirit of biocapitalism” in which responsibility for social welfare is displaced onto individuals' ability to act as enterprising subjects [20]. Reflecting many of the core beliefs at the center of the overlap between survival prepping and resistance to neoliberalism, this narrative links the shame or stress of living with perpetual contingency to skepticism about the connections between medical institutions' motivation and financial gain. It exposes the contradiction at the heart of current “self-care” logics, in which individuals demonstrate their personal virtue by understanding and managing their somatic condition through responsible consumption practices [20]. Strikingly, fish mox users reinforce this logic of self-care even as they voice critique of it. Consuming fish mox emerges as both a pragmatic response to lack of adequate medical care and resistance to medical institutions' expertise and claims to care—it constitutes a resistant form of self-care.

## 4. Analysis: communicability of fish mox as populist expertise

While Briggs' discussion of communicability stresses the power of official institutions to define and manage public health [5] his work speaks to the broader phenomena in which information is transformed into authoritative *knowledge* through alignment with a community's core and collective beliefs. Narratives about people's experiences thus reflect shared expectations about how broad sociopolitical and economic institutions influence individual health and well-being and are verified as fact through experiential confirmation. As such, narratives that contest official or mainstream knowledge constitute a kind of populist expertise about the safety and efficacy that is legitimately grounded in communities' experiences with powerful social institutions. They draw upon three related core propositions about contemporary American social, political, and economic life that are confirmed by individuals' experience: (1) an expectation that pharmaceuticals act the same way on microbes in animal and human bodies; (2) a skepticism of professional expertise and institutional distrust; and (3) a deep belief that pharmaceutical and medical institutions are key to the perpetration of health-based inequalities. Spanning survivalist future-oriented narratives and contemporary accounts of precarious poverty, these beliefs reflect specific ways that distrust and unreliability of “expert” institutions influences health-related behaviors.

### 4.1. Pharmaceutical similarity and self-experimentation

While every package of fish mox includes a caution that the pills are intended only for *ornamental* fish treatments and not for human consumption, online users question whether this is a meaningful distinction. Some tongue-in-cheek circumvent one expected line of challenge by noting that fish mox does not lead to morphological changes; as one user concludes her defense of the practice: “And no I didn't grow scales or gills” (survivalistboards.com). More often, users demonstrate a kind of defense of one of global health's central premises post Pasteur (i.e. bodies are bodies and germs are germs regardless of context), now extended past the species line. However, rather than making the case that fish and human bodies are functionally analogous as is considered by scholarship on multispecies ethnography, this narrative posits the ontological stability of microbial life (and its counters). If dogs, cats, fish, birds, and humans are all vulnerable to similar strains of bacterial infection and all treated with broad-spectrum antibiotics, it is rational to expect similarity in substance. Fish mox users thus position themselves as expert consumers by demonstrating that the treatments are materially equivalent: “they look and smell the same as their ‘human’ counterparts and from what I understand they are manufactured in the same places as the human ones” (studentdoctor.net). By placing human in scare quotes this user rhetorically dismisses as meaningless species-level distinctions in bodily care.

Distrusting corporate disclaimers, online communities participants engage in a kind of crowd-sourced self-experimentation. Some extensively track the results of using fish mox to treat the same symptoms for which medical professionals had previously prescribed medication (i.e. chronic bronchitis or sinus pain). Their reports of such self-experimentation emphasize their carefulness matching dosing between past prescriptions for common ailments and fish mox manufacturers' labels. They similarly stress the ease of self-diagnosis for common ailments—which is proven by the absence of symptoms after fish mox treatment—and downplay or avoid any discussion of side effects. Others share the results of their extensive comparison research—detailing color patterning, manufacturing marks, and dosage standards and, occasionally, basic chemical composition analysis that they do themselves. Participants rely on attaching their findings to the socially legitimizing discourses of evidence-based rationality to establish their conclusions as authoritative and themselves as populist experts. For example, this user's contribution to a threaded discussion on drugs.com about whether fish-mox and human are the same product:

“I found a definitive answer that satisfied me that fish mox is safe for humans. The marking on the side of Thomas' Fish Mox Forte is ‘AA 825’. When you look that up on Drugs.com's Pill Identifier cite [sic] it identifies it as amoxicillin - for humans. Check it out [link deleted]. It does not mention anything about the product being for fish. This convinces me that the fish and human versions are identical, for that brand at least (drugs.com September 2016).”

Characterizing the manufacturer's self-report as a “definitive” answer, the user lays out their expectations for a proof that bypasses the regulatory institutions that define one product for humans and the other for fish and goes directly to the source. Here users' expectation that antibiotics (and microbes) work similarly in bodies across the species line is proven by crowd-sourced research. This kind of crowd-sourced experimentation is central to fish mox communicability. Once users credibly accept that “mox is mox”, their attention focuses on questions of purity and dosage safety. Both of these require trust in the established medical experts who embody the institutions that make, sell regulate, and dispense these drugs. The absence of this trust is a common theme in discussions of fish mox.

### 4.2. Institutional distrust

Users are acutely aware that their belief that fish mox is efficacious and safe directly counters the recommendation of the medical community, possibly even to the point of illegality. Yet, they shift the locus of distrust back onto health institutions and challenge their authority to define fish mox as dangerous by producing alternatively expert claims. Such challenges anticipate why medical professionals caution against fish mox—especially concerns about population level drug overuse—and argue that populist use of fish mox is not materially different than institutional use. As one user notes in an aside in the middle of their defense of fish mox consumption in constrained circumstances:

“I also understand fears about making more drug resistant bacteria, but let's call a spade a spade, the medical establishment has been and continues to be more of a danger to create and cause drug resistant bacteria then a few fringe people treating themselves incorrectly (studentdoctor.net Nov 2014).”

This user's skepticism that “a few fringe people” taking fish mox are worse for public health than established medical practice demonstrates an understanding of the evidentiary bases of institutional claims against populist pharmaceutical experimentation. By contesting these claims on their own terms, this critique bestows crowd-sourced experimentation with the legitimacy and trust it in turn strips from the medical establishment. Fish mox narratives thus express and reinforce the community's fundamental mistrust of contemporary health institutions and governmental safety regulations by characterizing them as in the service of corporations and self-interest and thus are less credible sources of knowledge than distributed populist expertise.

This institutional mistrust is unsurprising in a context of communities that feel left behind by the very institutions designated as “caring” ones, and it manifests in suspicion of the role financial interest plays in establishing medical standards. The following description of how a user assessed the different knowledge claims about fish mox illustrates a narrative of communicability that links financial self-interest to credibility:

“All sorts of Americans out there on the web are talking about Fish Mox for human consumption. Plenty of them claim to use it when they need an antibiotic, and say it works. I have always told my college freshmen students that blogs and forums are bad sources, and it's true that I don't know anything about any of the bloggers. The thing that strengthens my sources is the sheer volume of firsthand accounts of people who say they took fish amoxicillin and got better. I have to trust that they are all telling the truth, because why would someone lie about taking fish medication? What would they have to gain? The pharmaceutical companies, on the other hand, who say not to trust the fish meds, stand to lose a lot (sic) money (Blue Lake Review, March 2015).”

This account grants users' experience more legitimacy than established institutions on the basis of an imagined weighing of each one's financial self-interest. It mirrors a core survivalist belief about self-reliance and responsibility that cautions against overreliance in social, political, or medical institutions, while shifting them to ultimately indict such organizations rather than praising the independent individual as in classic prepper narratives. This ultimately supports the legitimacy of populist expertise as trustworthy due to its connection to users' dire straits while delegitimizing pharmaceutical corporations and regulatory apparatus due to its connection to profit margins. This is at once a condemnation of the role of health institutions in late-stage capitalism and a celebration of the experimental resourcefulness of those left behind by it. Cultural narratives about consuming fish mox thus circulate in a sphere of communicability that grants individual experiential knowledge truth status and that questions the authority of established health institutions on the basis of how communities experience injustice.

### 4.3. Institutions and inequalities

Closely tied to mistrust in the motives and authority of formal health institution is a belief that the medical establishment's warning against fish mox creates, or at least perpetrates, the systemic inequalities that lead people to consume fish mox. This expresses a fundamental understanding that health care in the United States is a matter of politics and economics rather than care—an argument whose broad outlines many medical professionals may share [21]. Two key elements to this narrative link fish mox consumption to American precarity: (1) nostalgia for a lost period of social and economic stability and (2) suspicion of alliances between pharmaceutical and medical institutions. They convey a kind of outrage that adults in the United States rely on fish mox by characterizing this practice as shameful. Relying on a doubled nature of shame (i.e. individuals feel shame taking fish mox, but the real shame indicts social and political structures), this ultimately indicts medical institutions broadly for perpetrating health-based inequalities and is thus extended to other health-related behaviors.

The first element of this critique suggests that over past decades medical *care* has decreased, implicitly linking this inversely to the escalating certainty in future disaster that characterizes prepper communities. As this user explains, fish mox consumption is both a reflection of poor care and a solution to it:

“Most clinics and HMOs seem to treat their patients like cattle and follow a scripted treatment regime rather than actually thinking and tailoring the treatment to the individual. My wife has experienced a number of times and we really dislike recent medical practices, it's really gone downhill [sic] in the past 20 years. It's no longer a service when you have to argue with the person you're paying for the service.”

“Now that I'm aware of the fish antibiotics, I will shave off countless wasted hours and grief going through their gatekeeper system. It's a shame that as adults we have to resort to such methods (survivalistboards.com June 2011).”

This user's reliance on nostalgia for a lost past both suggests that medical institutions are no longer necessary sites of care and that medical professionals' role is one of gatekeeping rather than healing. Drawing on a belief in populist expertise that distributes credibility away from these established authorities, this narrative can be persuasive. Many medical professionals would probably agree with this person's first two sentences, but argue that the target and solution of such “shame” should not undercut their legitimacy.

Users draw on an abundance of observable details to make claims about secret and nefarious linkages that produce credible narratives about fish mox's connection to inescapable realities of health-based inequalities much as in the Warao at the center of Briggs and Mantini-Brigg's analysis [10]. This endorsement of fish mox in a discussion about poverty on reddit is emblematic of such narratives:

“But if you need antibiotics and are uninsured, there IS a way you can get them. You don't have to die, just because you can't afford to pay some dick in a labcoat [sic] thousands of dollars to help finance his new yacht (reddit.com December 2011).”

This user relies on publicly observable details about the increasing unaffordability of basic medication, the rise of pharmaceutical industry profits over the last decades, and generally increased economic stratification to suggest that fish mox regulations are part of a larger plot against impoverished communities. Such skepticism illustrates a deeply held narrative that medical institutions' themselves are an active and intentional barrier to public health.

## 5. Conclusions

Systematic analysis of fish mox users' narratives about their consumption reveals that they mirror deeply American cultural logics about inequality and medical institutions and are rendered authoritative knowledge via populist expertise. As individuals experience a world in which they feel left behind, fish mox emerges as both critique and panacea. According to the above cultural logics and as evinced by crowd-sourced experimentation, fish mox *should* be safe for humans: (1) If the crowd-sourced investigations into manufacturing practices and the user-reports successful healing are correct, it makes sense to conclude no difference between fish- and human-grade supplements; (2) If neoliberalism has taught users they are individually responsible for health outcomes, it makes sense that users are more concerned with their own care and recovery than worries about global drug resistance; (3) And (most especially), if communities experience medical or pharmaceutical institutions as unable to heal chronic bodily inequality, it makes sense to trust a populist expertise that offers to redistribute financial benefits back to impoverished communities.

These are all logical extensions of individuals' experiences of health, wellbeing, and inequality. They implicate fish mox in a broader schema of populist expertise that challenges the legitimacy of expert institutions' ability to produce authoritative knowledge. Cultural narratives about fish mox mirror those that challenge vaccine use [7],[11] by establishing new forms of expertise over health and risk.

Significantly, populist expertise about fish mox does not challenge the legitimacy of public health knowledge practices, *per se*, but rather its institutional ability to use this knowledge in the service of the care it promises. The same cultural logics are employed to both justify and caution against fish mox consumption. This means that public health educators will be more successful taking seriously the multiple social and economic variables that contribute to individuals' decisions to consume fish mox. These beliefs reflect a context of increasing economic (and correlated health) stratification such that stocking up on fish mox becomes a way to care for oneself in the absence of formal care institutions. Justifications to stock up on fish mox reflect and support narratives about institutions' contributions to this stratification. Rather than direct resistance to public health *qua* public health, they instead challenge institutions' definitions of what constitutes pharmaceutical expertise. This is analogous to Sobo's findings about vaccine resistant parents, which offers a model for how successful public health interventions can account for in-group “cultural cognition” [7]. Following, it is incumbent on public health interventions to address these root causes of fish mox consumption, to take these cultural narratives seriously as forms of populist expertise about contemporary health and continual precarity, and to demonstrate to communities how they advocate for system-level changes. While it is improbable that fish mox consumption is a purely American practice, it is worth considering how fish mox's credibility—as a form of critique of established medicine and solution to its failures—emerges from the specifics of American public health and will only be magnified by current public health politics.

## Footnotes

1 There are a number of broad-spectrum fish antibiotics with slightly different names (Aqua Mox, Amoxfin, Fish Flex, etc.). Fish Mox is a particular brand manufactured by Thomas Labs as well as the generic term for these supplements, as used here.

2 A 2002 concerned letter to the New England Journal of Medicine by Drs. Goff, Koff, and Geiling [15] supports this origin story.

3 While all of the narratives presented here were gathered from publicly available websites, I have removed identifying information including pseudonyms of the user statements I quote.

4 While fish mox often costs more per pill than human amoxicillin for people with health insurance, going to the doctor involves co-pays, transportation costs, and the requires ability to take time off work.

5 The frequency of tooth pain in these narratives as catalyst for using fish mox is striking, and most probably reflects the separation of dental care from general health care in the United States, which leads to a higher population of underinsured in the former.
